# Effect of extracellular polymeric substances on the colony size and morphological changes of *Microcystis*

**DOI:** 10.3389/fpls.2024.1367205

**Published:** 2024-03-05

**Authors:** Jiaxin Pan, Zhongyong Yang, Nan Hu, Bangding Xiao, Chunbo Wang, Xingqiang Wu, Tiantian Yang

**Affiliations:** ^1^ College of Hydraulic and Envrionmental Engineering, China Three Gorges University, Yichang, China; ^2^ Key Laboratory of Algal Biology of Chinese Academy of Sciences, Institute of Hydrobiology, Chinese Academy of Sciences, Wuhan, China; ^3^ School of Envrionmental Studies, China University of Geosciences, Wuhan, China; ^4^ Kunming Dianchi and Plateau Lakes Institute, Dianchi Lake Ecosystem Observation and Research Station of Yunnan Province, Kunming, China

**Keywords:** extracellular polymeric substances, *Microcystis*, colony formation, colony size, morphology

## Abstract

Surface blooms of colony-forming *Microcystis* are increasingly occurring in aquatic ecosystems on a global scale. Recent studies have found that the *Microcystis* colonial morphology is a crucial factor in the occurrence, persistence, and dominance of *Microcystis* blooms, yet the mechanism driving its morphological dynamics has remained unknown. This study conducted a laboratory experiment to test the effect of extracellular polymeric substances on the morphological dynamics of *Microcystis*. Ultrasound was used to disaggregate colonies, isolating the cells and of the *Microcysti*s suspension. The single cells were then re-cultured under three homologous EPS concentrations: group CK, group Low, and group High. The size, morphology, and EPS [including tightly bound EPS (TB-EPS), loosely bound EPS (LB-EPS), bound polysaccharides (B-polysaccharides), and bound proteins (B-proteins)] changes of colonies were closely monitored over a period of 2 months. It was observed that colonies were rapidly formed in group CK, with median colony size (*D_50_*) reaching 183 µm on day 12. The proportion of colonies with a size of 150–500 µm increased from 1% to more than 50%. Colony formation was also observed in both groups Low and High, but their *D_50_* increased at a slower rate and remained around 130 µm after day 17. Colonies with a size of 50–150 µm account for more than 50%. Groups CK and Low successively recovered the initial *Microcystis* morphology, which is a ring structure formed of several small colonies with a *D_50_* of 130 µm. During the recovery of the colony morphology, the EPS per cell increased and then decreased, with TB-EPS and B-polysaccharides constituting the primary components. The results suggest that colony formation transitioned from adhesion driven to being division driven over time. It is suggested that the homologous EPS released into the ambient environment due to the disaggregation of the colony is a chemical cue that can affect the formation of a colony. This plays an important but largely ignored role in the dynamics of *Microcystis* and surface blooms.

## Graphical Abstract

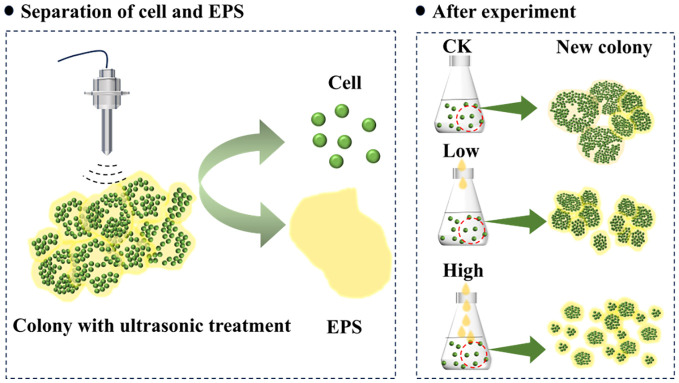

## Highlights

- The TB-EPS and B-polysaccharides primarily contribute to colony formation.
- *Microcystis* unicell forms a colony by cell adhesion.
- *Microcystis* achieves specific morphotypes by rapid cell division.
- Homologous EPS has an inhibitory effect on the morphological recovery of *Microcystis.*

## 1 Introduction

*Microcystis* spp. are widespread, harmful bloom-forming cyanobacteria (Sun et al., 2016; Chen et al., 2020; Kondo et al., 2023). Its colonies, characterized by individual cells embedded within extracellular matrices rich in extracellular polymeric substances (EPS) (Xiao et al., 2018), are ecologically significant for *Microcystis* enhancing persistence of individual cells in dynamic environments. Through cell aggregation, colonies provide protection, efficient resource uptake, and create unique microenvironments (Stal, 2017; Xiao et al., 2018). These advantages bolster ecological success and shape community structure (Visser et al., 2005; Yamamoto et al., 2011).

Under natural conditions, *Microcystis* mainly exists in colonies. But in typical laboratory cultures, it exists as single cells. Laboratory *Microcystis* strains can form colonies due to various biotic and abiotic factors. Biotic factors, such as EPS (Omori et al., 2019; Wei et al., 2020, 2021), *Microcystin* (Gan et al., 2012), allelopathy (Li et al., 2020), and zooplankton (Yang and Kong, 2012), are commonly involved. Abiotic factors include Ca^2+^ (Chen and Lurling, 2020; Huang et al., 2022), Mg^2+^ (Omori et al., 2019), temperature (Wei et al., 2020), and light (Wei et al., 2021). For *Microcystis* in the field, colony size may become larger under the disturbance caused by different wind and wave conditions (Duan et al., 2019). However, under some environmental stresses, colonies may also disaggregate to adapt to the novel environment. Strong turbulence can cause the disaggregation of a colony (Wu et al., 2019b). In late autumn and winter, as light decreases and water temperatures drop, *Microcystis* colonies in the lake may disaggregate resulting in a decreasing size (Tsujimura et al., 2000).

Colony formation is achieved through the mechanisms of cell division and adhesion, and these two mechanisms are often interactive (Xiao et al., 2017) posing a challenge in evaluating their respective roles. Division and adhesion are usually quantified by comparing colony cell growth with total cell number growth (Xiao et al., 2017; Duan et al., 2018). Studies have shown that after colony-induced culturing of laboratory *Microcystis* strains and field isolated single-celled *Microcystis* strains, the newly formed colonies did not exhibit specific types of morphology in a few months (Duan et al., 2018; Chen and Lurling, 2020). When future cell division events take place, the arbitrary arrangement of cells within the colony becomes regular (Otsuka et al., 2000; Sun et al., 2016). It helps to form colonies with distinct morphological characteristics.

The formation of a colony is seemingly associated with EPS. For example, EPS has been found to contribute to the adhesion, cohesion, and aggregation of *Microcystis* cells providing the foundation for the development of multi-cellular structures (Karampatzakis et al., 2017; Liu et al., 2018). This matrix is excreted by *Microcystis* into their surrounding environment (Duan et al., 2018), where it functions as a critical scaffold for colony formation. EPS can be categorized as soluble EPS (SL-EPS) and binding EPS (B-EPS) according to the degree of tight binding with a colony, and B-EPS is further divided into loosely bound EPS (LB-EPS) and tightly bound EPS (TB-EPS) (Omori et al., 2019; Tan et al., 2020). The main constituents of *Microcystis* EPS are polysaccharides and proteins, and many humic acid-like components have been identified recently (Xiao et al., 2019; Van Le et al., 2022). In natural water bodies, EPS can be released by disaggregation of a colony and death of a cell (Sigee et al., 2007). However, how EPS affect the colony dynamics of *Microcystis* has remained unclear.

To enhance the understanding of colony formation and dynamics, the single-celled *Microcystis* was used to culture a colony with homologous EPS in the laboratory. In the present study, ultrasound was used to simulate the natural forces that cause the disaggregation of a colony and to isolate the cells and EPS. By incubating the single cells with varied amount homologous EPS, we aim to (i) explore the effect of EPS on the morphological characteristics of *Microcystis* and (ii) investigate the mechanism of colony formation during this process. This study is expected to contribute to a better understanding of the differences between single-celled and colonial morphologies of *Microcystis*, the strategies involved in community formation, and the role of EPS in *Microcystis* colony formation and morphological changes.

## 2 Materials and methods

### 2.1 *Microcystis* colony culture and collection

On 20 March 2023, a phytoplankton net with a mesh size of 63 µm was used to collect a thin layer of surface scum composed of cyanobacteria that was collected during the early stages of a spring bloom in Guanqiao fish pond located in Wuhan, China. The collected cyanobacterial colonies were quickly transported to the laboratory. They were immediately filtered through a 300-µm mesh filter followed by a 45-µm mesh filter to eliminate coarse impurities. The filtered and concentrated cyanobacterial colonies were subjected to a pre-culturing process in 10% BG11 medium, at a temperature of 25°C, under a light/dark cycle of 12 h/12 h with light exposure of 25 µmol photons m^−2^ s^−2^, for a duration of 3 days (Zhao et al., 2019; Gao et al., 2020; Xu et al., 2023). Morphological identification was conducted, which revealed the predominance of *Microcystis* species (**Supplementary Figure S1C**).

### 2.2 Acquisition of cells and homologous EPS of *Microcystis*

To isolate cells from *Microcystis* colonies, the *Microcystis* colonies were subjected to pretreatment using an Ultrasonic Processor (VCX150, Sonics & Materials Inc., USA). The colonies were dispersed into individual cells (**Supplementary Figures S2B** and **S3B**) through the application of ultrasonic waves (Wu et al., 2012; Zhang et al., 2021). The ultrasonic amplitude was set at 50%, and a total energy of 108–112 J was released over a 4-min duration. The single cells obtained from ultrasonic dispersion were stained with fluorescein diacetate (FDA) at 100 µg mL^−1^ and subsequently kept in darkness for 5 min (Chen et al., 2005). Upon exposure to an excitation light source, the cells exhibited a vibrant green fluorescence (**Supplementary Figure S3B**) confirming that the ultrasonic treatment employed did not induce cellular mortality (Yang et al., 2021). Both the cells and EPS were isolated by centrifugation at 9,000 rpm for 10 min. The experimental cells were obtained by subjecting them to three rounds of resuspension and centrifugation in 10% sterile BG-11 medium.

The resulting supernatant from centrifugation at 9,000 rpm was collected and subsequently filtered using GF/F membranes (0.22 µm; Waterman, UK) to obtain *Microcystis* EPS that was devoid of cells. This collected EPS is called homologous EPS. To prevent the EPS from rapidly deteriorating at ambient temperature, the separated homologous EPS was promptly refrigerated and stored for later use.

### 2.3 Experiment design

The homologous EPS collected during pre-treatment was added to cells at different concentrations, determined by the dissolved organic carbon (DOC) concentration. This resulted in EPS concentrations of 0 mg L^−1^ (CK), 0.66 mg L^−1^ (Low), and 12 mg L^−1^ (High) within the culture system (**Supplementary Table S1**). During the collection of *Microcystis* in the field, the pond’s DOC content was measured to be 12 mg L^−1^ at a depth of 0.1 m below the water surface.

All groups were cultured in Erlenmeyer flasks containing 10% sterile BG11 medium. In all groups, the starting cell density was consistent at 6.5×10^8^ cell L^−1^. The experiments were conducted in an Illuminated Incubator (PGX-100A-LED, Jiangsu Tianling Instrument Co., Ltd., Yancheng, China). Culture conditions mirrored those of wild *Microcystis* colonies, with gentle agitation provided by manual shaking two to three times per day (Gao et al., 2020; Xu et al., 2023). Other culturing conditions were the same as those in Section 2.1. Samples were collected every 3 days during the initial phase when cells reaggregated into microcolonies and every 5 days when the size of reaggregated colonies stabilized. Nutrient concentrations within the culture system were monitored throughout the sampling period to ensure adequate nutrient availability. All experimental groups were conducted in quadruplicate, and the results were reported as averages.

### 2.4 Growth parameters and morphology

The *Microcystis* population is enumerated after the alkaline hydrolysis using an optical microscope (BX43, Olympus Corporation). The relative growth rate (ν) of *Microcystis* was calculated as follows (Xu et al., 2023):


$$\text{ν}=\left(C_{tj}-C_{ti}\right)/C_{ti}$$

where *C_ti_* and *C_tj_* (cells L^−1^) are the cell densities of *Microcystis* at two consecutive sampling times.

Chlorophyll a (Chl*a*) was measured using a 4.8-mL sample. The Phytoplankton Analyzer (Phyto-PAM, Walz Co., Erlangen, Germany) was utilized for the assessment of *Microcystis* photosynthetic activity (Ye et al., 2023). The Fv/Fm parameter represents the potential maximum conversion efficiency of *Microcystis* photosystem II, as it is overwhelmingly dominant (Wang et al., 2018). Photos were taken using software (HaoKangBioImaging) on a microscope.

### 2.5 Zeta potential, pH, and dissolved oxygen

The zeta potential of *Microcystis* was measured with a Malvern Zetasizer (Malvern-Nano-ZSMalvern, UK) (Tattibayeva et al., 2022). The pH was determined with a pH meter (pH100A, EcoSence, China). Additionally, the dissolved oxygen (DO) in the *Microcystis* fluid was quantified using a YSI instrument (YSI Pro ODO, USA).

### 2.6 Colony size and compactness

The study utilized a Laser *In-Situ* Scattering and Transmissometer instrument (LISST-200X, Sequoia Scientific Inc., Bellevue, WA, USA) to measure colony size, cell size, and biovolume concentration. The median colony size (*D_50_*), representing the median biovolume concentration location, was used to characterize the diameter of *Microcystis* cells and colonies. Before measuring, the *Microcystis* sample was appropriately diluted with distilled water. Then, 10 mL of the diluted sample was used to determine the size distribution and biovolume concentration (*V_colony_* in µL L^−1^) of the original *Microcystis* colony. After the measurements, the *Microcystis* fluid was retrieved, and the colonies were disaggregated into single cells through alkaline hydrolysis at 85 °C for 6–8 min (Wang et al., 2015). The size distribution and biovolume concentration (*V_cell_* in µL L^−1^) of *Microcystis* cells were subsequently assessed using the LISST-200X. The volume ratio (VR) of the cells to colonies was calculated according to established laboratory protocols from prior studies (Wu et al., 2020; Xu et al., 2023), and it indicates the relative compactness of colonies:


$$VR=V_{cell}/V_{colony}$$

where *V_cell_* (µL L^−1^) and *V_colony_* (µL L^−1^) are the mean biovolume concentrations of single cells and of colonies in a sample.

### 2.7 EPS and DOC measuring

Coomassie Brilliant Blue G-250 and phenol-sulfuric acid were used to measure polysaccharides and proteins, respectively (Wang and Xing, 2009; Ma et al., 2021; Duan et al., 2022) in this study. The sample was first centrifuged at 5,000 rpm for 15 min. After removing the supernatant, the remaining sediment was resuspended using a 0.05% NaCl solution. The suspended algae solution was then centrifuged at 5,000 rpm for LB-EPS measurement (Xiao et al., 2018). The residual sediment was re-suspended with a 0.05% NaCl solution at an adjusted pH of 10 and heated at 45°C for 4 h. Subsequently, centrifugation was performed at 11,000 rpm for 15 min, and the resulting supernatant was collected for the measurement of polysaccharides and proteins (Yang et al., 2008; Xiao et al., 2012, 2019). LB-EPS encompasses loosely bound proteins and polysaccharides. TB-EPS comprises tightly bound proteins and polysaccharides.

The content of dissolved polymeric substances (S-EPS) was represented by DOC in this study. The DOC in liquid samples filtered through GF/F membranes was quantified using a Total Organic Carbon Analyzer (TOC-VCPH, Shimadzu Co., Kyoto, Japan).

### 2.7 Statistical analysis

SPSS statistics software (version 27, Chicago, IL, USA) was used to determine the differences in physiological regulation of *Microcystis* at different periods of colony formation and the differences in colony formation caused by homologous EPS. Values with *p<* 0.05 were considered significant, while those with *p<* 0.01 were considered very significant. All data images were produced using Origin 2021 software (OriginLab, Northampton, MA, USA).

## 3 Results

### 3.1 Growth of *Microcystis* under different EPS concentrations

**Figure 1:**
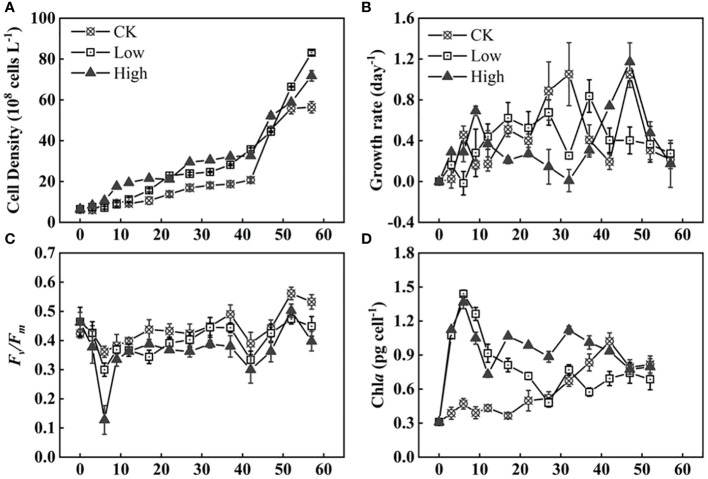
Time series of cell density **(A)**, growth rate **(B)**, photochemical efficiency of PSII (Fv/Fm) **(C)**, and Chl*a* concentration **(D)** of *Microcystis* under three homologous EPS concentrations (**Supplementary Table S1** for explanation of the experimental groups).

As shown in **Figure 1A**, there was no significant difference in *Microcystis* cell density during the first 0–6 days. However, the cell density in group High remained consistently higher than the other two groups for the following 42 days. In **Figure 1B**, the growth rate of group CK had a small peak of 0.46 day^−1^ during 3–6 days followed by a small decrease and a rise to the second peak of 1.05 day^−1^ during 27–32 days. This pattern repeated, reaching the third peak of 1.05 day^−1^ during 42–47 days. During the final 20 days of the experiment, the rapid increase in growth rate of group CK ceased and then decreased to a stagnant growth state. It showed a relatively stable and low cell density curve in **Figure 1A**. The growth rate of group Low rose and fell during the experiment in general. But it decreased to 0.25 day^−1^ between days 27 and 32 before peaking at 0.84 day^−1^ in the following 5 days. Group High had higher growth rate at the beginning and end of experiment. Specifically, the peak values observed were 0.69 and 1.17 day^−1^ on days 6–9 and 42–47. At the same time, it was observed that the dissolved total phosphorus (DTP) in this set of systems was almost depleted by the end of day 12, and the medium was replenished in a timely manner (**Supplementary Figure S4**). The growth rate of group High reached a second small peak of 1.7 on days 22–27. A third peak in growth rate was observed after 47 days. The growth rate of group Low reached a small peak on day 22 and increased rapidly to 7.4 on days 32–52. In the long term, the addition of homologous EPS is beneficial to *Microcystis* population.

The Chl*a* per cell in group CK was significantly lower than that in group Low (*p<* 0.05). In comparison to group Low, the difference in Chl*a* content in group High was much more significant (*p*< 0.01). The Chl*a* content per cell increased during the initial 6 days, followed by a decrease as the days progressed, and eventually stabilizing at a relatively constant value. The Chl*a* content was similar among the different treatment groups (**Figure 1D**).

During the first 6 days, the photochemical efficiency of PSII (Fv/Fm) significantly decreased in all treatment groups. The magnitude of the decrease was directly proportional to the amount of added homologous EPS. The group with high homologous EPS addition showed a reduction of 0.34, which was two to five times lower than the low addition group and group CK (**Figure 1C**). Overall, group CK had the highest Fv/Fm value, while the group with high homologous EPS addition had the lowest value.

### 3.2 Changes in physicochemical parameters of the *Microcystis*

**Figure 2:**
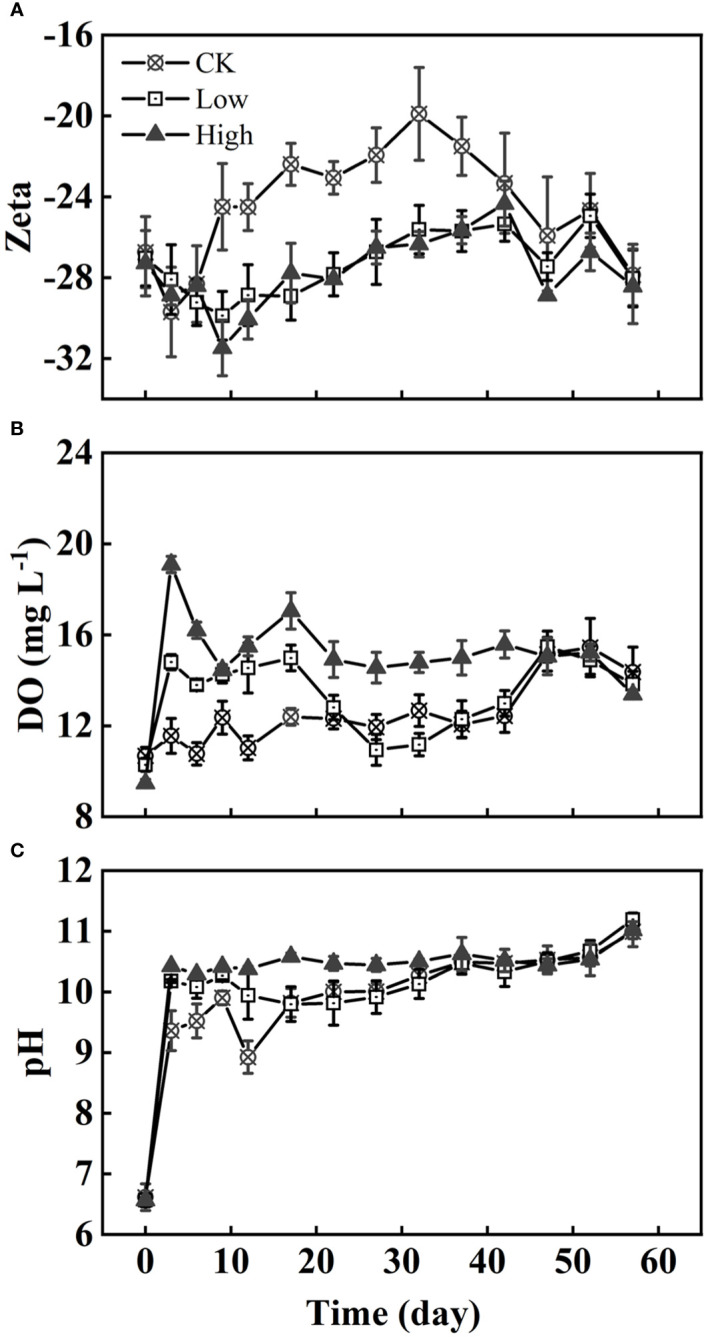
The variation of the Zeta potential **(A)**, DO **(B)**, and pH **(C)** of algae under three homologous EPS concentrations over time. Difference between group CK and two EPS addition groups on Zeta potential and DO were very significant and significant (*p*< 0.01, *p<* 0.05).

It was observed that the absolute value of the Zeta potential in group CK increased in the first 0–3 days before decreasing. Groups Low and High experienced a decrease in the absolute value of Zeta potential until 12 days (**Figure 2A**). From days 10 to 40, the absolute value of the Zeta potential of group CK remained at 20 to 24, which was lower than that of the other two groups. There is no significant difference in the Zeta potential between group Low and group High. The concentration of DO and pH of group High were higher than those in group Low. Similarly, group Low had higher DO and pH than group CK in the first 20 days (**Figures 2B, C**).

### 3.3 The impact of EPS concentration on colony size and morphology

#### 3.1.1 Dynamics of colony morphology

**Figure 3:**
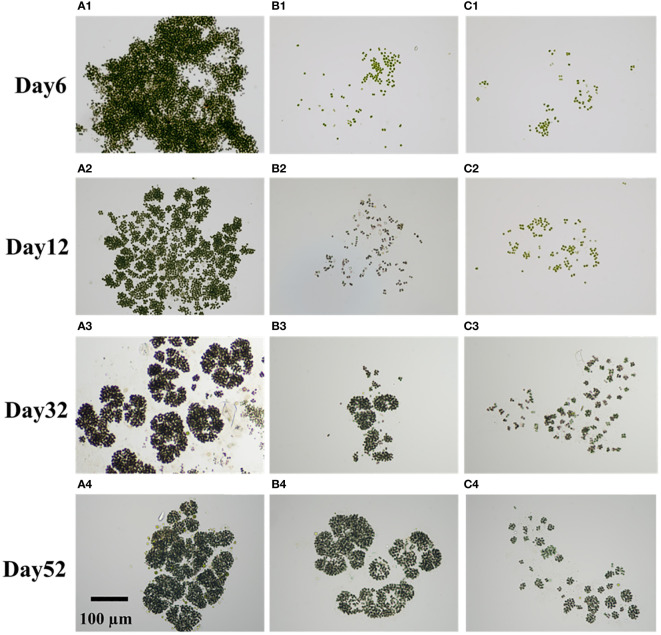
Different colony forms formed over time. They are microphotographs taken at ×20 magnification [group CK: groups **(A1–A4)**; group Low: **(B1–B4)**; High: **(C1–C4)**]. Row 1: day 6; row 2: day 12; row 3: day 32; row 4: day 52.

As shown in **Figure 3**, the addition of homologous EPS had a lasting and profound effect on the restoration of colony morphology. Both groups CK and group Low successfully returned to the original *Microcystis novacekii* form by the end of the experiment (**Figures 3A4, B4**). However, the colonies of group High consist of small spherical or nearly spherical colonies (diameter<50 µm) forming loose large colonies (**Figure 3C4**). For group CK, some large colonies were observed on day 6. On day 12, many gaps appeared between the colonies, which were then divided into distinct and tiny blocks (**Figure 3A2**). By day 27, the colonies had taken on the classic form of *Microcystis novacekii*, with three to five small colonies connected in rings (**Figure 3A3**). The differences between group Low and group CK were that there were fewer large colonies in the process of morphological recovery in group Low (**Supplementary Figure S5**), and the ring form formed by connecting small groups does not appear until day 32 (**Figures 3B1–B3**).

#### 3.1.2 The impact of EPS on colony size and compactness

**Figure 4:**
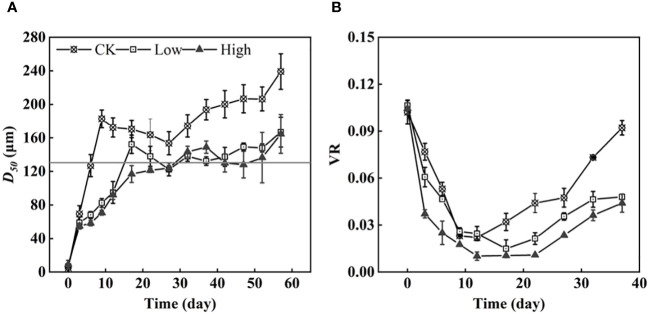
The medium-sized colonies under three homologous EPS concentrations and the original colonies (horizontal solid line: 130 µm) **(A)** and the colony volume ratio (VR) **(B)**.

During the first three days of the experiment, *D_50_* increased at the same rate in all groups. However, after that, group CK exhibited a greater growth trend in *D_50_*, reaching its first peak on the 12th day and gradually decreasing thereafter. In contrast, *D_50_* under different initial EPS concentrations remained relatively stable at approximately 120 µm on day 17 (**Figure 4A**). The maximum *D_50_* values of groups Low and High were 166 and 187 µm, respectively. The two groups of colonies were similar in size. *D_50_* of group CK and two EPS addition groups were significantly different (*p*< 0.05).

Simultaneously, it was discovered that different concentrations of EPS influenced the formation of *Microcystis* colonies. The colony size in group CK quickly recovered to the size of the original colonies (**Figure 4A**). Meanwhile, the cell density remained relatively constant during this period. The recovery speed of colony size in groups Low and High was slower than that of group CK, requiring 17 and 27 days, respectively. During the initial stages of the experiment, the colonies VR decreased due to loose cell adhesion (**Figure 4B**). In **Supplementary Figure S5**, the proportion of the VC for 0–50, 50–150, and 150–500 µm in the total VC was roughly the same on day 0. Specifically, the VC proportion for 0–50 µm was 72% ± 4%, the VC proportion for 50–150 µm was 27.5% ± 3.5%, and the VC proportion for 150–500 µm was 1%. After 3 days, the VC proportion for 0–50 µm decreased in each group, while the VC proportion for 50–150 µm increased significantly, and the VC proportion for 150–500 µm increased slightly. After 12 days, the VC proportion for 150–500 µm in group CK remained stable at over 50%. The VC proportion for 50–150 µm also remained stable at more than 38% ± 4%, while the VC proportion of 0–50 µm accounted for only a very small percentage of 3%–5%. Compared with group CK, colonies measuring 150–500 µm accounted for the majorities of the proportion. Two groups with added homologous EPS accounted for more colonies of 50-150 µm proportion. Additionally, the proportion of 50–150 µm in group High was higher than that in group Low.

Group High exhibited the lowest *D_50_* indicating that a high concentration of homologous EPS had an inhibitory effect on the process of colony formation when compared with no and low concentrations of homologous EPS. After 12 days, there was a significant increase in cell density, with a rise of (10.4 ± 2.4) × 10^8^ cells L^−1^, and the cell density began to increase. Although group CK had the lowest cell density, it exhibited the fastest VR growth. This indicates that the EPS produced by cells during cultivation was more effective in promoting cell density than the added homologous EPS.

### 3.4 Changes in EPS of *Microcystis* colonies

**Figure 5:**
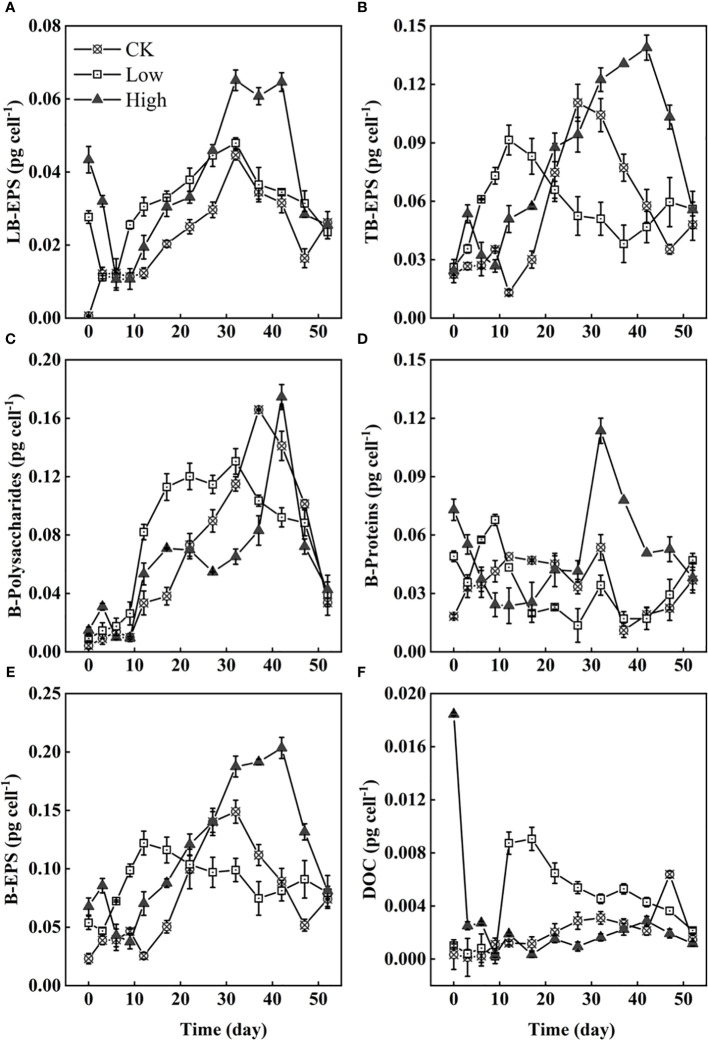
The content of LB-EPS **(A)**, TB-EPS **(B)**, B-polysaccharides **(C)**, B-protein **(D)**, bound extracellular polymers (B-EPS) **(E)**, and DOC **(F)**. All on individual *Microcystis* cells.

In this experiment, the EPS of *Microcystis* were analyzed by their content of polysaccharides and proteins as representative structural EPS. The ultrasonic treatment used to extract the EPS was not entirely thorough, as evidenced by the incomplete extraction of TB-EPS (**Figure 5B**). The addition of homologous EPS resulted in a rapid response from *Microcystis* cells, as indicated by the production of LB-EPS. The strength of the positive response was directly proportional to the amount of homologous EPS added (**Figure 5A**). Initially, the added homologous EPS mainly consisted of soluble EPS, with a smaller proportion of LB-EPS. Among them, proteinaceous substances had a higher proportion than polysaccharides (**Figures 5A, B, D, F**). During the experiment, the LB-EPS on individual cells in both groups Low and High first decreased from 0 to 6 days and then increased. In groups CK, Low, and High, the LB-EPS content on individual cells reached its highest levels at approximately 32 days, with values of 0.045, 0.048, and 0.065 pg cell^−1^, respectively (**Figure 5A**). In contrast to the immediate decrease in LB-EPS content in groups CK and Low, group High maintained its peak levels for 10 days. Over the course of 0–20 days, the TB-EPS on individual cells in group Low was significantly higher than in groups CK and High. In **Figure 5B**, the TB-EPS peak values were 0.092, 0.111, and 0.139 pg cell^−1^ for groups Low, CK, and High, respectively. The peak order of B-polysaccharides on cells was similar to that of TB-EPS, with only group High showing a peak of up to 0.114 pg cell^−1^ at 32 days. According to the trend of extracellular substances on cells from 0 to 6 days, polysaccharides are suggested to be mainly TB-EPS, while proteins are suggested to be mainly LB-EPS (**Figures 5A–D**). The trends in B-EPS on individual cells in each group were generally similar, with group CK maintaining lower B-EPS levels than the other two groups (**Figure 5A**), resulting in the formation of denser *Microcystis* colonies in group CK. The DOC level in group CK consistently remained low, with a slight decrease after a gradual increase (**Figure 5F**). In group Low, the DOC level was above 0.003 pg cell^−1^ after the ninth day and continued to decline after approximately 20 days. The DOC level in group High rapidly decreased from 0.018 to 0.003 pg cell^−1^ within the first 3 days and has since fluctuated between 0 and 0.003 pg cell^−1^.

## 4 Discussion

Under no and low homologous EPS addition, *Microcystis* cells isolated from field strains can recover to the colonies with original morphological level, including colony size and morphology. Based on VR, it suggests that the formation of *Microcystis* colonies from single cells to stable shapes involves two main stages. Stage 1: Cells aggregate into small colonies through adhesion (decreasing VR). Stage 2: Colonies become more compact (increasing VR) and eventually form a specific colony morphology.

At the beginning of stage 1, the Chl*a* content of cell and DO all increased (**Figures 1**, **2**). The pH rapidly increased to 9–10 within 3 days (**Figure 2**). Such alkaline condition could be created by the photosynthesis of cyanobacteria. The colony’s photosynthesis is promoted by the elevated pH, which increases until it surpasses 10 (Fang et al., 2018). This could facilitate colony formation (Bano and Siddiqui, 2004; Fang et al., 2018). At stage 1, which lasted for about 12 days, the cell densities of all three groups did not increase significantly (**Figure 1A**), while colonies were observed in all groups. Particularly, in group CK, the colony size of *Microcystis* on the sixth day is comparable to original colonies (**Figure 4A**). Therefore, this process of cell aggregation is most likely caused by adhesion. Although adhesion has been considered as one of the mechanisms for the formation of colonies (Duan et al., 2019), the involved processes have not been fully clarified. Colonies formed in all groups suggest that the cell adhesion is not caused by homologous EPS. Contrarily, the results suggest that homologous EPS can inhibit this process. The more homologous the EPS added, the stronger the inhibition observed (**Figure 3**). Under different homologous EPS additions, the rate of change in *D_50_* in the first 12 days was group CK > group Low > group High (**Figure 3**). At the same time, counterintuitively, it was found that the VR of colonies did not become dense with the decrease in *D_50_*. Group CK with a larger size had the largest colony VR (**Figure 3**). The TB-EPS on cells was not completely removed (**Figure 5B**). Most *Microcystis* (>85%) have a layer of hydrophobic proteins outside the cell wall named S-layer, which is important for cell adhesion and surface recognition (Šmarda et al., 2002; Schachtsiek et al., 2004; Zu et al., 2020). This structure may overlap with TB-EPS, which allows cells in group CK to directly recognize and adhere to special substances between TB-EPS, thus forming larger and relatively denser colonies. A recent study (Duan et al., 2022) shows that few perssads of LB-EPS in *Microcystis novacekii* are enhanced in favor of colony adhesion. Cells with low and high EPS concentrations first face the binding of TB-EPS to homologous EPS, then resulting in looser colonies. Besides, due to the larger physiological differences between different strains (Zhang et al., 2007; Wu and Song, 2008; Du et al., 2023), adhesion of *Microcystis* occurs in the same morphological type relating to different EPS components (Duan et al., 2022).

Stage 2 occurred after 12 days. Contrary to single-celled *Microcystis* strains forming colonies in the laboratory, colony B-EPS secretion capacity was inversely related to colony size (Xu et al., 2016). The organic matter synthesized by cells is first used for proliferation rather than self-protection especially during the last period of rapid cell growth. Consequently, a reciprocal limitation is observed between cell growth and the produce of EPS. Generally, group CK exhibited significantly larger colony sizes, although with the lowest recorded cell density and absolute Zeta potential values. It is easier to form a bloom with a lower absolute value of cell surface potential resulting in larger colony size of the bloom (Cao and Yang, 2010; Liu et al., 2016). In all groups, the absolute value of Zeta potential increases first and decreases, which is roughly consistent with the time of B-EPS change (**Figures 2A**, **5E**). It suggests that the change in surface zeta potential over time may be caused by changes in the composition and content of B-EPS (Bernhardt et al., 1985; Liu et al., 2016).The colony of *Microcystis novacekii* has an orderly arrangement of cells, and it is spherical or nearly spherical, and three to five small colonies are connected into a ring structure (Yu et al., 2007). Through microscopic observation, we found that the colonies appeared to have a more obvious morphological type in the period of rapid growth of cell density, which we identified as *Microcystis novacekii* (groups CK and High) (**Figure 3**). Cell division results in the orderly arrangement of cells within the colony (Xiao et al., 2017). Group High may take longer to exhibit this pattern. Based on this finding, we hypothesize that in the natural environment, the homologous EPS, which is often accompanied by colony disaggregation, is a signaling important chemical cue that transmits environmental stress to *Microcystis* inhibiting the formation of new colonies.

It is reported that co-occurring microorganisms isolated from *Microcystis* blooms from Lake Taihu can increase or decrease *Microcystis aeruginosa* colony size (e.g., *Chryseobacterium* sp. and *Bacillus cereus*) (Wu et al., 2019a). In this experiment, the possible role of the co-occurring microorganisms in the formation of the colony remains uncertain. In the process of colony formation, EPS on cells always increases and then decreases. The TB-EPS and B-polysaccharides were more abundant (**Figure 5**) indicating that they may contribute to colony maintenance more than LB-EPS, S-EPS, and B-proteins. It is undeniable that more EPS is conducive to colony formation, but the colony that has formed a morphological structure seems to maintain the morphological structure with a low EPS presence (**Figure 5**). The vital role of EPS in the dynamics of *Microcystis* colonial morphology, as revealed by this study, implies that monitoring the EPS content in lakes may be another potential means for early warning of harmful cyanobacterial blooms.

## 5 Conclusion

The colony-forming process of *Microcystis* from single cells’ specific morphological characteristics is helpful to further understand the early development process of *Microcystis* blooms. During the colony formation, adhesion contributed more. Then cell division took over, accompanied by the increase in EPS on *Microcystis*. The EPS decreased when the *Microcystis* was divided into a morphologically distinct structure. The homologous EPS has a certain influence on the development of colony morphology features. This study provides new insights into the single-cell to colonial transformation of *Microcystis* and the processes associated with colony morphological changes.

## Data availability statement

The original contributions presented in the study are included in the article/**Supplementary Material**. Further inquiries can be directed to the corresponding author.

## Author contributions

JP: Data curation, Formal analysis, Methodology, Software, Supervision, Visualization, Writing – original draft, Conceptualization, Investigation, Project administration, Validation, Writing – review & editing. ZY: Funding acquisition, Writing – review & editing. NH: Investigation, Writing – review & editing. BX: Resources, Writing – review & editing. CW: Funding acquisition, Writing – review & editing. XW: Supervision, Writing – review & editing. TY: Conceptualization, Data curation, Formal analysis, Funding acquisition, Investigation, Methodology, Project administration, Supervision, Writing – original draft, Writing – review & editing.
