# Supplementary material for: Effect of extracellular polymeric substances on the colony size and morphological changes of Microcystis
Source: Front Plant Sci. 2024 Mar 5;15:1367205. doi: 10.3389/fpls.2024.1367205 (PMC10948609; doi:10.3389/fpls.2024.1367205)
Supplement: Supplementary file 1 [file Presentation_1.pdf]

Supplementary materials

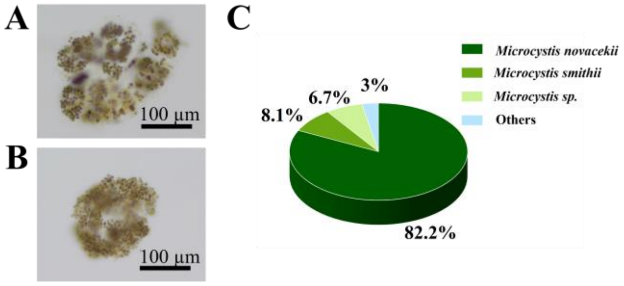

Figure S1 Microscope images of the dominant *Microcystis novacekii* **(A**, **B)** and composition of algal samples **(C)**.

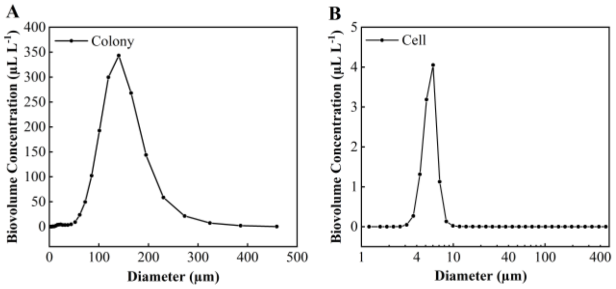

Figure S2 *Microcystis* colony size distribution before ultrasonic treatment **(A)** and cell size distribution after ultrasound treatment **(B)**.

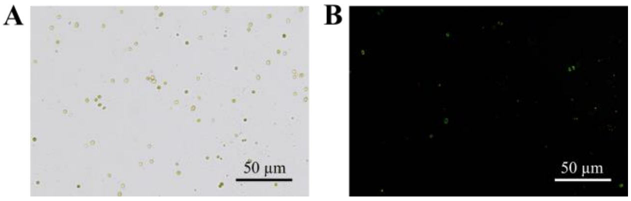

Figure S3 Cells after ultrasound treatment **(A)** and cells after FDA staining **(B)**.

Table S1 Initial homologous EPS addition and the EPS concentration of the system

Concentration/ Volume	CK		Low		High
EPS concentration (mg L^{-1})	0	0.012	0.12	1.2	12
EPS volume (mL)	0	0.076	0.92	9.16	91.6
10% BG-11(mL)	777	777.9	776.1	767.8	685.4
Cell liquid (mL)	123	123	123	123	123
System volume (mL)	900	900	900	900	900

Note: The experiment was conducted with 5 different initial homologous EPS addition levels. For better analysis, the EPS concentration of 0 mg L^{-1} and 0.012 mg L^{-1} were collectively recorded as group CK, which considered to be 0 mg L^{-1}. The EPS concentration of 0.12 mg L^{-1} and 1.2mg L^{-1} was combined as the group Low, and the

average concentration of the two groups was taken to be the initial concentration. And that is 0.66 mg L^{-1}.

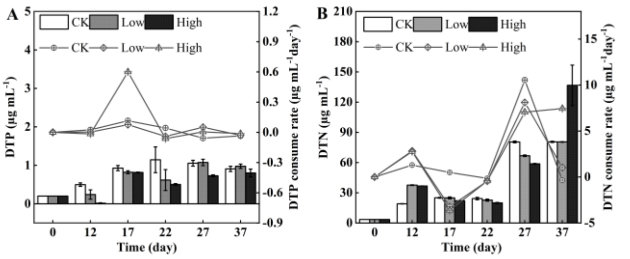

Figure S4 Dissolved total phosphorus (DTP) (**A**), dissolved total nitrogen (DTN) (**B**) in algal fluid and their corresponding consumption rates. In 10% BG-11 culture medium, DTP is 0.2 μg L^{-1} and DTN is 0.35 μg L^{-1}. In the consumption rate of DTP and DTN, positive values represent nutrients release and negative values represent nutrients consumption.

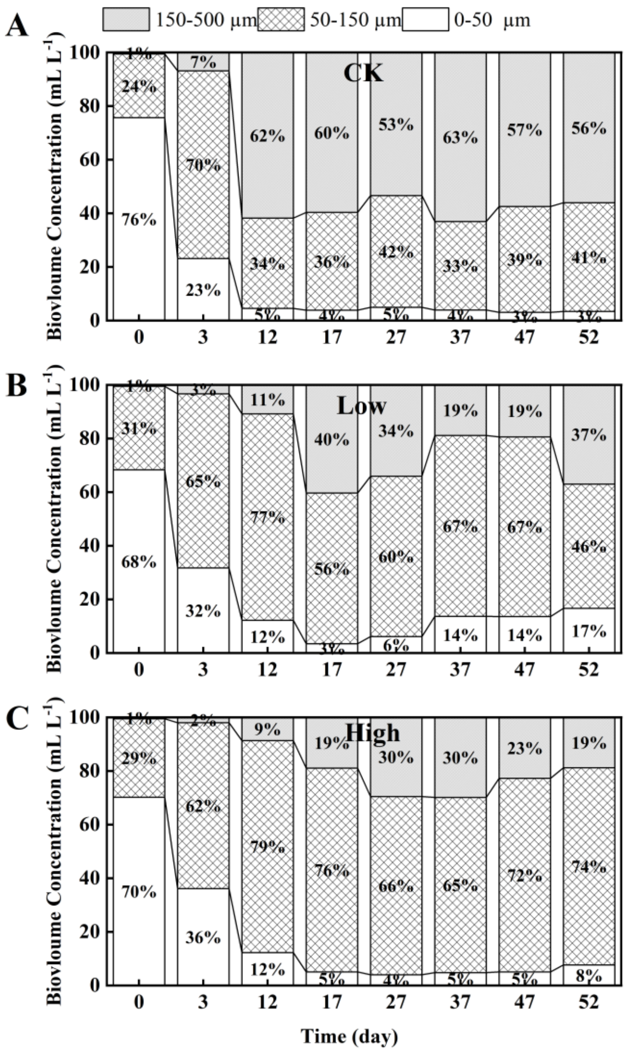

Figure S5 The proportion of biovolume concentration of different colony size groups (0-50 μm, 50-150 μm, 150-500 μm) to total biovolume concentration of group CK **(A)**,

group Low **(B)** and group High **(C)**.
